# Iodoxybenzoic Acid Supported on Multi Walled Carbon Nanotubes as Biomimetic Environmental Friendly Oxidative Systems for the Oxidation of Alcohols to Aldehydes

**DOI:** 10.3390/nano8070516

**Published:** 2018-07-10

**Authors:** Bruno Mattia Bizzarri, Issam Abdalghani, Lorenzo Botta, Anna Rita Taddei, Stefano Nisi, Marco Ferrante, Maurizio Passacantando, Marcello Crucianelli, Raffaele Saladino

**Affiliations:** 1 Department of Ecology and Biology, University of Tuscia, Largo dell’Università, 01100 Viterbo (VT), Italy; bruno004@hotmail.it (B.M.B.); lorenzo.botta@unitus.it (L.B.); 2 Department of Physical and Chemical Sciences, University of L’Aquila, Via Vetoio, I-67100 Coppito (AQ), Italy; esam.ghani1988@hotmail.com (I.A.); maurizio.passacantando@aquila.infn.it (M.P.); 3 Great Equipment Center Electron Microscopy Section, University of Tuscia, Largo dell’Università, 01100 Viterbo (VT), Italy; artaddei@unitus.it; 4 Laboratori Nazionali del Gran Sasso, Via G. Acitelli, 22, 67100 Assergi (AQ), Italy; stefano.nisi@lngs.infn.it (S.N.); marco.ferrante@lngs.infn.it (M.F.); 5 Trace Research Centre, Via I. Silone 6, 64015 Nereto (TE), Italy

**Keywords:** IBX, supported IBX, MWCNTs, gold, oxidation, aromatic alcohols

## Abstract

Iodoxybenzoic acid (IBX) supported multi walled carbon nanotube (MWCNT) derivatives have been prepared as easily recyclable solid reagents. These compounds have been shown to be able to mimic the alcohol dehydrogenases and monooxygenases promoted oxidation of aromatic alcohols to corresponding aldehydes. Their reactivity was found to be dependent on the degree of functionalization of MWCNTs as well as from the chemical properties of the spacers used to bind IBX on the surface of the support. Au-decorated MWCNTs and the presence of longer spacers resulted in the optimal experimental conditions. A high conversion of the substrates and yield of desired products were obtained.

## 1. Introduction

The oxidation of alcohols to corresponding carbonyl compounds is one of the most fundamental and important processes in synthetic organic chemistry. Although a variety of methods and reagents have been developed, they all suffer from the difficulty of selectively oxidizing primary alcohols to aldehydes without the concomitant formation of carboxylic acids and other over-oxidation products [1]. The oxidation of alcohols to aldehydes is usually performed in the presence of stoichiometric reagents [2] including the Dess–Martin oxidation [3], the Swern and Corey-Kim reaction [4], and the Burgess reagent [5]. Heavy metal reagents have been also used in catalytic procedures, for instance, hydrogen-transfer reactions (Ru, Rh, Ir) [6], and Oppenauer oxidations (Al, Zr, lanthanides) [4]. On the other hand, metal-free oxidations are desired processes in the context of green-chemistry due to the known toxicity and high environmental impact of metal species. In this context, biotechnological applications of oxidative enzymes, e.g., alcohol dehydrogenases and monooxygenases with high environmental compatibility, have been widely evaluated [7]. Unfortunately, there are drawbacks related to the use of these enzymes in large scale applications, encompassing the necessity to regenerate Nicotinamide Adenine Dinucleotide NAD(P)^+^ for alcohol dehydrogenases, and the use of low molecular weight redox mediators (such as 2,2,6,6-tetramethylpiperidin-1-yl-oxidanyl (TEMPO) and 1-hydroxybenzotriazole (HOBt)) in the case of monooxygenases [8]. For example, the “coupled substrate approach”, proposed for the regeneration of NAD(P)^+^ through the use of ketones and aldehydes as co-substrates, has shown close equilibrium conditions (that is low conversion of the substrate), complex purification procedures, and enzyme inhibition [9]. Alternative procedures, namely the “coupled enzyme approach”, where the NAD(P)^+^ regeneration is performed by a second enzyme, are disadvantaged by the complex realization of two-enzyme kinetic processes and by the exclusive enzyme specificities for the substrate and co-substrate [10]. Thus, continuous interest is devoted to developing biomimetic and environmentally friendly metal-free solid supported reagents for the selective oxidation of alcohols that are able to mimic a biological material in its structure or function [11]. 

In the last few years, solid-supported ortho-iodoxybenzoic acid (1-hydroxy-1λ5,2-benziodoxol-1,3-dione, IBX) reagents able to convert primary alcohols to corresponding aldehydes, have been prepared by the immobilization of the active iodine species on chemically inert supports including silica [12], polystyrene beads [13], and ionic liquids [14]. These reagents have been reported to be biomimetic [15,16], contemporary solving problems associated with insolubility in common organic solvents and low stability towards moisture of IBX, combining the environmental advantages of simple recyclability of the reagent, simple purification procedures, easier reaction optimization, and safety related issues [13,17]. For these reasons, IBX supported reagents have been recognized as environmental friendly reagents [18,19]. Single layer two-dimensional sp^2^ carbons graphene oxide (GO) has been also successfully applied as a solid support for the immobilization of IBX [20]. 

Multi Walled Carbon Nanotubes (MWCNTs) consist of several layers of graphene sheets rolled up into a cylindrical shape surrounding a central tube with lengths in the micrometer scale and diameters up to 100 nm [21]. They offer a high surface area for loading of the active species as well as biocompatibility and mechanical resistance [20]. Notably, the reactivity of supported active species can be tuned, moving from GO to MWCNTs as a consequence of the local curvature and of the specific physical and chemical properties of the support [22]. Here, we describe the preparation of IBX supported MWCNT solid reagents as biomimetic and environmentally friendly oxidative systems. Two different types of supports were investigated, MWCNTs and, an alternative, Au-decorated MWCNTs. The direct formation of amide (spacer-mediated) linkages with IBX and the high binding affinity of sulfur containing IBX derivatives for the Au surface were both applied for the immobilization of the iodine active reagent. The novel IBX supported MWCNT systems showed high reactivity and selectivity in the oxidation of primary alcohol to corresponding aldehydes, the efficacy of the system being controlled by the nature of the support and the length of the spacer.

## 2. Materials and Methods 

### 2.1. Materials

Alcohols **1**–**8**, organic solvents, HAuCl_4_, NaBH_4_, and GH Polypro membrane filters were purchased from Sigma-Aldrich (Saint Louis, MO, USA) and used without further purification. Gas chromatography mass spectrometry (GC–MS) was recorded on a Varian 410 GC-320 MS (Palo Alto, CA, USA) using a VF-5 ms column (30 m, 0.25 mm, 0.25 µm), and an electron beam of 70 eV. All experiments were done in triplicate. Ultrapure HNO_3_ and HCl obtained from a sub-boiling system (DuoPUR, Milestone, Bergamo, Italy) and ultrapure 18.2 MΩ water from a Milli-Q (Millipore, Burlington, MA, USA) were used for the sample dissolution. X-ray photoelectron spectroscopy (XPS) and inductively coupled plasma mass spectrometry (ICP–MS) were performed through an ultrahigh vacuum PHI 1257 system and an Agilent 7500 ICP–MS instrument under clean room ISO6 (Santa Clara, CA, USA), respectively.

### 2.2. Preparation of oxMWCNTs I

In a round-bottomed flask, equipped with an egg-shaped magnetic stirring bar, MWCNTs and a mixture of concentrated H_2_SO_4_–HNO_3_ (3:1) were stirred for 4.0 h at r.t. and an additional 12 h at 40 °C. The reaction mixture was cooled down to r.t. and cold H_2_O (400 mL) was poured into the reactor. The mixture was washed by centrifugation at 4000× *g* rpm (30 min), and the supernatant was removed. The remaining solid was further washed with deionized H_2_O (200 mL). At each washing step, the mixture was centrifuged (4000 rpm for 30 min), filtered using GH Polypro membrane filters 0.2 μm and the supernatant was removed. The resulting oxidized MWCNTs (oxMWCNTs **I**) were dried in vacuo and used without further purification.

### 2.3. Preparation of Oxidizing Solid Reagents IV A–B 

oxMWCNTs **I** were suspended in Dimethyl Formamide DMF (0.8 mg/mL) and treated with *N*,*N*-diisopropylcarbodiimide (DIC; 250 mg, 2 mmol), HOBt (270 mg, 2 mmol), and *N*,*N*-diisopropyl ethylamine (DIPEA; 700 μL, 4 mmol) in a round-bottomed flask with an egg-shaped magnetic stirring bar for 15 min. Thereafter, the appropriate diamine (1,2-di-aminoethane for **IIA** and 1,6-diaminoethane for **IIB**) (2.0 mmol) was added to the solution under stirring for 12 h at 30 °C. The resulting solution was washed with DMF (5 × 5.0 mL) by centrifugation (4000× *g* rpm, 20 min) and filtered using GH Polypro membrane filters 0.2 μm. The resulting NH_2_–MWCNTs **II A**–**B** were dried in vacuo. Successively, NH_2_–MWCNTs **II A**–**B** (200 mg) suspended in DMF (0.8 mg/mL) were treated with DIC (790 mg, 6 mmol), and DIPEA (2.1 mL, 12 mmol), in a 500 mL round-bottomed flask with an egg-shaped magnetic stirring bar. Thereafter, 2-Iodo Benzoic Acid IBA (1.5 g, 6.0 mmol) was added to the solution and the mixture stirred for 8 h at 30 °C. The resulting IBA–MWCNTs **III A**–**B** were washed with DMF and H_2_O by centrifugation (4000× *g* rpm, 20 min) and filtered using GH Polypro membrane filters 0.2 μm. IBA–MWCNTs **III A**–**B** were then suspended in H_2_O (125 mg/250 mL) in a round-bottomed flask, and added to Oxone^®^ (950 mg, 1.5 mmol) and methane sulfonic acid (100 μL, 1.5 mmol) under stirring for 8 h at r.t. Thereafter, IBX–MWCNTs **IV A**–**B** were washed with DMF (5 × 5.0 mL) and H_2_O (3 × 5.0 mL) and filtered using GH Polypro membrane filters 0.2 μm. 

### 2.4. Preparation of Oxidizing Solid Reagents VIII A–B

MWCNTs (100 mg) were sonicated in 100 mL of ethanol for 2 h. Afterwards, 8.5 mL of 0.1 M HAuCl_4_ ethanolic solution was added. In order to obtain Au particles, reduction with 300 mg of NaBH_4_ was carried out by stirring for about 30 min. Then, Au–MWCNTs **V** was isolated by centrifugation and filtered using GH Polypro membrane filters 0.2 μm washed several times with ethanol and dried at 80 °C. 2-amino-1-ethanethiol (for NH_2_–Au–MWCNTs **VI A**) and 6-amino-1-hexanthiol (for NH_2_-Au-MWCNTs **VI B**) was dissolved in a mixture of water (20 mL) and 1.0 M HCl (3.0 mL). Au–MWCNTs **V** (30 mg) and ethanol (3.0 mL) were added and the mixture was left under magnetic stirring for 24 h. After that time, the product was isolated by centrifugation, washed three times with 0.01 M NaOH and ethanol, and filtered using GH Polypro membrane filters 0.2 mm. Resulting NH_2_–Au–MWCNTs **VI A**–**B** were dried under argon stream. NH_2_–Au–MWCNTs **VI A**–**B** (200 mg) were suspended in DMF (0.8 mg/mL) and treated with DIC (790 mg, 6 mmol) and DIPEA (2.1 mL, 12 mmol) in a 500 mL round-bottomed flask with an egg-shaped magnetic stirring bar. Thereafter, IBA (1.5 g, 6 mmol) was added to the solution, and the mixture was stirred for 8 h at 30 °C. The resulting IBA–Au–MWCNTs **VII A**–**B** were washed with DMF and H_2_O by centrifugation (4000× *g* rpm, 20 min) and filtered using GH Polypro membrane filters 0.2 μm. IBA–Au–MWCNTs **VII A**–**B** were suspended in H_2_O (125 mg/250 mL) in a round-bottomed flask, then Oxone^®^ (950 mg, 1.5 mmol), and methane sulfonic acid (100 μL, 1.5 mmol) were added and stirred for 8 h at r.t. Thereafter, IBX–Au–MWCNTs **VIII A**–**B** were washed with DMF (5 × 10 mL) and H_2_O (3 × 10 mL) and filtered using GH Polypro membrane filters 0.2 μm. 

### 2.5. Preparation of Oxidizing Solid Reagent VIII–C

11-mercapto-1-undecanol was dissolved in a mixture of water (20 mL) and 1.0 M HCl (3.0 mL). Au–MWCNTs **V** (30 mg) and ethanol (3.0 mL) were added and the mixture was left under magnetic stirring for 24 h. After that time, the product was isolated by centrifugation, washed three times with 0.01 M NaOH and ethanol, and filtered using GH Polypro membrane filters 0.2 mm. The resulting OH–Au–MWCNTs **VI C** was dried under argon stream. OH–Au–MWCNTs **VI C** (200 mg) was suspended in DMF (0.8 mg/mL) and treated with DIC (790 mg, 6 mmol), and DIPEA (2.1 mL, 12 mmol) in a 500 mL round-bottomed flask with an egg-shaped magnetic stirring bar. Thereafter, IBA (1.5 g, 6.0 mmol) was added to the solution and the mixture stirred for 8 h at 30 °C. The resulting IBA–Au–MWCNTs **VII C** was washed with DMF and H_2_O by centrifugation (4000× *g* rpm, 20 min) and filtered using GH Polypro membrane filters 0.2 μm. IBA–Au–MWCNTs **VII C** was suspended in H_2_O (125 mg/250 mL) in a round-bottomed flask, then Oxone^®^ (950 mg, 1.5 mmol) and methane sulfonic acid (100 μL, 1.5 mmol) were added and stirred for 8 h at r.t. Thereafter, IBX–Au–MWCNTs **VIII C** was washed with DMF (5 × 10 mL) and H_2_O (3 × 10 mL) and filtered using GH Polypro membrane filters 0.2 μm.

### 2.6. Transmission Electron Microscopy (TEM), Scanning Electron Microscopy (SEM), and X-Ray Photoelectron Spectroscopy (XPS) Analyses

For transmission electron microscopy (TEM), samples were suspended in bi-distilled water. Droplets of sample suspensions (10 µL) were placed on formvar–carbon coated grids and allowed to adsorb for 60 s. Excess liquid was removed gently by touching the filter paper. Samples were observed with a JEOL 1200 EX II electron microscope (Waltham, MA, USA). Micrographs were acquired by the Olympus SIS VELETA CCD camera equipped with iTEM software (Waltham, MA, USA). For scanning electron microscopy (SEM), the sample suspensions (50 µL) were let to adsorb onto carbon tape attached to aluminum stubs and air dried at 25 °C. The observation was made by a JEOL JSM 6010LA electron microscope (Waltham, MA, USA) using Scanning Electron (SE) and Back Scattered Electrons (BSE) detectors. Energy Dispersive Spectroscopy (EDS) analysis was carried out to reveal the chemical elements. X-ray photoelectron spectroscopy (XPS) analysis was done in an ultrahigh vacuum PHI 1257 system equipped with a hemispherical analyzer, operating in the constant pass energy mode (with the total energy resolution of 0.8 eV) and using a non-monochromatized Mg Kα radiation source. The distance between the sample and the anode was about 40 mm, the illumination area was about 1 × 1 cm^2^, and the analyzed area was 0.8 × 2.0 mm^2^ with a take-off angle between the sample surface and the photoelectron energy analyzer of 45°. The energy scale was calibrated with reference to the binding energy of the C 1s at 284.8 eV with respect to the Fermi level. Survey scans of the III–B, IV–B, VII–A, and VIII–A compounds acquired in the range of 0–1100 eV (not shown here) displayed the contribution coming from the main elements involved in the reaction process for all of the samples: carbon, nitrogen, oxygen, sulfur, gold, and iodine. No contaminant species were observed within the sensitivity of the technique.

### 2.7. Inductively Coupled Plasma Mass-Spectrometry (ICP–MS) Analysis

The samples were weighed (from 1.6 to 6.9 mg) and transferred in Fluorinated ethylene propylene (FEP) vials, previously washed to avoid any kind of external contamination. Regia solution was chosen for the mineralization as it combines the oxidizing capacities of HNO_3_ with the complexing capacities of chlorides against I_2_ produced during digestion. In particular, 750 µL of HCl and 150 µL HNO_3_ were added and the solution was heated to 80 °C for 3 hours. The volume was adjusted to 5.0 mL and then diluted another 10 times before the ICP–MS analysis. The analysis was performed with an Agilent 7500 ICP–MS instrument (Palo Alto, CA, USA). Four standards at 10, 20, 50, and 100 ppb of iodine and gold were used for calibrating the instrument.

### 2.8. Oxidation of Aromatic Alcohols 

The oxidation of alcohols **1**–**8** (1.0 mmol) in EtOAc (10 mL) was performed by adding the appropriate solid reagent (**IV A**–**B** or **VIII A**–**C**, 1.2 eq) to a single neck round-bottomed flask equipped with a water condenser under magnetic stirring at reflux conditions (c.a. 80° C) for 24 h. At the end of the oxidation, **IV A**–**B** and **VIII A**–**C** were filtered off using GH Polypro membrane filters 0.2 μm and washed with EtOAc (5 × 10 mL). The yield of aldehydes **9**–**16** was determined by GC–MS analysis using n-dodecane (0.1 mmol) as an internal standard. The reactions were performed in triplicate. GC–MS was performed using a VF-5ms column (30 m, 0.25 mm, 0.25 µm) through the following program: injection temperature 280 °C, detector temperature 280 °C, gradient 50 °C for 2 min, and 10 °C/min for 60 min, flow velocity of the carrier (helium), 1.0 mL min^−1^. In order to identify the structures of the products, two strategies were followed. First, the spectra of identifiable peaks were compared with commercially available electron mass spectrum libraries such as that of National Institute of Standards and Technology (NIST-Fison, Manchester, UK). In this latter case, spectra with at least 98% similarity were chosen. Secondly, GC–MS analysis was repeated using commercially available standard compounds. The original mass spectra of compounds **9**–**16** are reported in Figure S1 (Supporting Information). 

## 3. Results

### 3.1. Preparation of IBX Supported MWCNTs and MWCNTs–Au Oxidizing Solid Reagents 

**Scheme 1:**
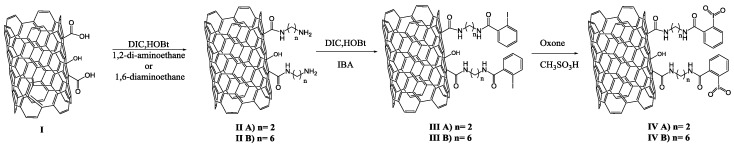
Preparation of IBX supported MWCNTs oxidizing solid reagents **IV A**–**B.**

The immobilization of IBX on MWCNTs was first based on the formation of an amide-type linkage between the spacer functionalized MWCNTs and 2-iodobenzoic acid (IBA), followed by activation of IBA to IBX (Scheme 1). In particular, commercially available MWCNTs were oxidized with HNO_3_/H_2_SO_4_ to oxMWCNTs **I** with the aim of increasing the amount of polar moieties (alcoholic and acidic groups) on the surface [23]. Next, ox-MWCNTs **I** was functionalized with selected alkyl diamino spacers (1,2-di-aminoethane and 1,6-diaminoethane) by coupling with *N*,*N*-diisopropyl carbodiimide (DIC) and 1-hydroxy benzotriazole (HOBt) in DMF at room temperature for 24 hours to yield the intermediates **II A**–**B**. The effectiveness of the coupling procedure was confirmed by Fourier Transform Infrared Spectroscopy (FTIR) analysis for **II-A** as a selected example. In particular, the peak at 1649 cm^−1^, corresponding to the stretching vibration of the carboxylic groups in oxMWCNTs **I** (Figure S2), was shifted to 1633 cm^−1^ in **II-A** as a consequence of the amide formation, in accordance with data previously reported for the functionalization of MWCNTs (Figure S3) [24]. The intermediates **II A**–**B** were successively suspended in DMF and treated with IBA at room temperature for 24 hours in the presence of DIC and HOBt to afford IBA–MWCNTs **III A**–**B**. The formation of the novel amide linkage was again confirmed by the shift of the amide peak from 1633 cm^−1^ to 1627 cm^−1^ (Figure S4). Finally, **III A**–**B** were activated to IBX-MWCNTs **IV A**–**B** by reaction with Oxone^®^ and methansulfonic acid. In this latter case, only a slight shift of the amide peak toward 1606 cm^−1^ was observed (Figure S5) [20]. 

**Scheme 2:**
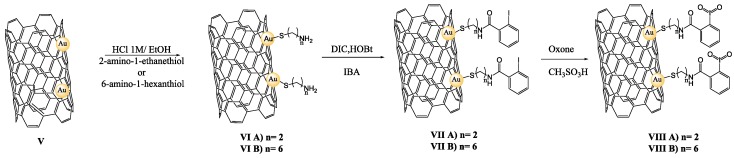
Preparation of Au decorated IBX supported MWCNTs oxidizing solid reagents **VIII A–B**.

**Figure 1:**
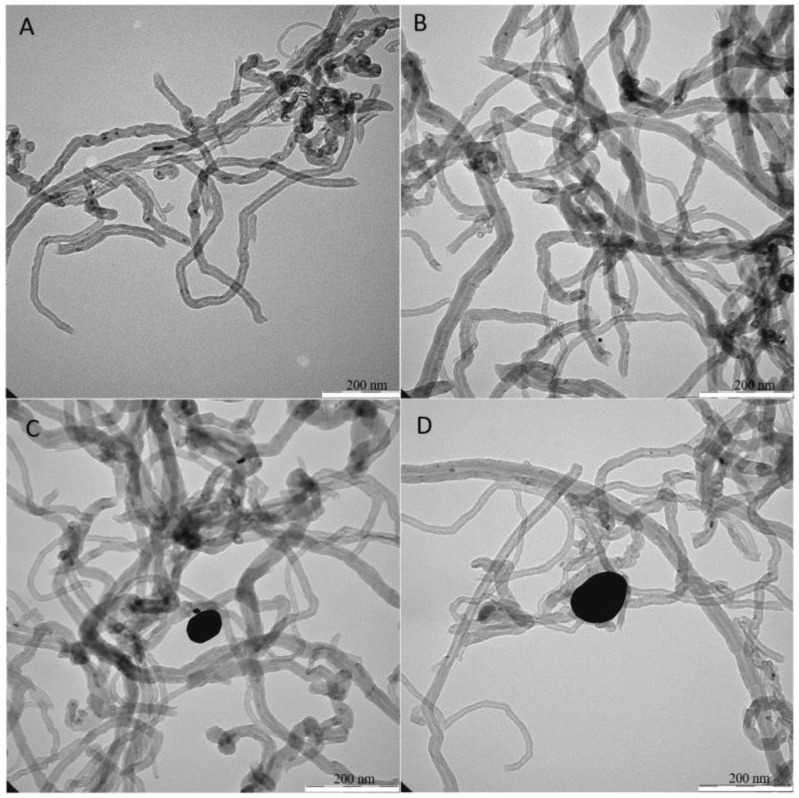
TEM images of **IV B** and **VIII B.** Panels A and C represent the oxMWCNTs and Au–MWCNTs after the loading procedure of IBX. Panels B and D represent **IV B** and **VIII B** recovered after the oxidation of alcohol **1.** In panels C and D, the black spot corresponds to the Au particle. (**A**) IBX-MWCNTs **IV B;** (**B**) IBX-MWCNTs **IV B** after the oxidation of alcohol **1**; (**C**) IBX–Au–MWCNTs **VIII B**; (**D**) IBX–Au–MWCNTs **VIII B** after the oxidation of alcohol **1.**

As an alternative, Au decorated Au–MWCNTs **V** were used instead of oxMWCNTs **I** as anchorage supports. Briefly, Au–MWCNTs **V** [25] were treated with selected alkyl mercapto-amino spacers (2-amino-1-ethanethiol and 6-amino-1-hexanthiol, respectively) in an acidic water/ethanol mixture (pH 2, HCl 1.0 M) to afford the intermediates NH_2_–Au–MWCNTs **VI A**–**B** by formation of covalent Au–sulfur bonds (Scheme 2). These intermediates were successively suspended in DMF and treated with IBA at room temperature for 24 h in the presence of DIC and HOBt to yield IBA–Au–MWCNTs **VII A**–**B**. Finally, IBX–Au–MWCNTs **VIII A**–**B** were obtained through the reaction of **VII A**–**B** with Oxone^®^ and methansulfonic acid [20]. The TEM images of **IV B** and **VIII B**, as the selected samples, are reported in Figure 1 (Panel A and C). In **VIII B**, the black-spots represent the Au particles, whose presence was unambiguously confirmed by SEM associated to BSE analysis (Figure S6). Note that the structural integrity of the MWCNTs was retained after the loading of IBX. 

**Scheme 3:**
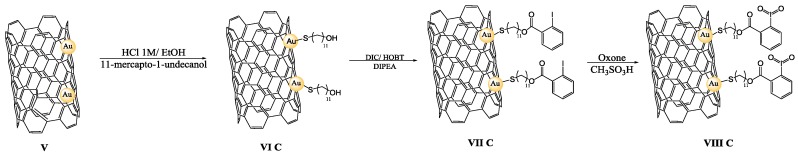
Preparation of Au decorated IBX supported MWCNTs oxidizing solid reagent **VIII C**.

Moreover, **VIII C** was prepared using a longer thio-alcohol spacer (11-mercapto-1-undecanol), with the aim to bind IBA through the formation of an ester bond instead of an amide bond (Scheme 3). Briefly, Au–MWCNTs **V** was treated with 11-mercapto-1-undecanol in HCl 1.0 M and EtOH to afford the intermediates **VI C** by formation of covalent Au–sulfur bonds (Scheme 3). This intermediate was successively treated with DIC, DIPEA, and IBA to yield **VII C**. Finally, **VII C** was suspended in H_2_O and treated with Oxone^®^ and methansulfonic acid to afford **VIII C**.

### 3.2. Determination of the Electron Binding Energies of the Elements by XPS Analysis

**Figure 2:**
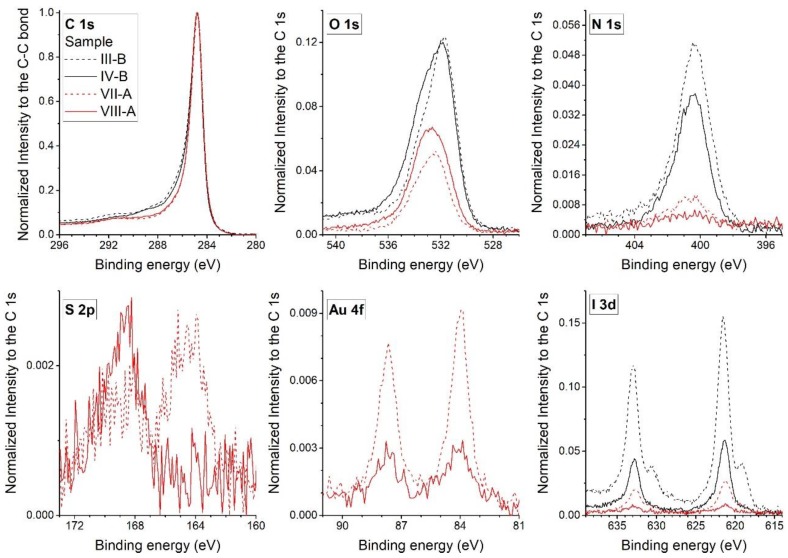
XPS of C 1s, O 1s, N 1s, S 2p, Au 4f, and I 3d core level spectra of **III B**, **IV B**, **VII A**, and **VIII A** compounds.

Figure 2 presents the detailed spectra of the C 1s, O 1s, N 1s, S 2p, Au 4f, and I 3d peaks of **III B**, **IV B**, **VII A,** and **VIII A**. All spectra were normalized to C 1s, which corresponded to the signal due to the MWCNTs support. In this way, we have the possibility of comparing the different peaks. XPS analysis clearly confirmed the presence of iodine and gold in the analyzed samples. Therefore, from the intensity of the XPS peaks (Figure 2) after the last step of the sample preparation (**III B** → **IV B** and **VII A** → **VIII A**), a slight leaching of Au and I was observed.

The C 1s spectra were fitted by the sum of five components assigned to C atoms belonging to: aromatic rings carbon (C=C/C–C, 284.8 eV), hydroxyl groups (C–OH, 285.9 eV), epoxy groups (C–O–C, 286.9 eV), carbonyl groups (C=O, 288.2 eV), and carboxyl groups (C=O(OH), 289.3 eV) (the hump at 290.6 eV was assigned to a π–π* shake-up satellite (in line with [20]). The O 1s spectra were fitted by the sum of three components: OH–C (533.4 eV), C–O–C (532 eV), and O=C (530.4 eV) [26]. Electron binding energies of the peak positions of N 1s, S 2p_3/2_, Au 4f_7/2_, and I 3d_5/2_ for all samples are listed in Table 1.

### 3.3. Determination of the Iodine Loading Factor by ICP–MS Analysis

The iodine Loading Factor (LF) for **IV A**–**B and VIII A**–**C**, defined as mmol of iodine per gram of support, was measured by Inductively Coupled Plasma Mass-Spectrometry (ICP–MS) analysis (Table 2). As reported in Table 2, **IV B** showed a Loading Factor (LF) significantly higher than **IV A** (entry 2 versus entry 1), highlighting the easier immobilization of IBA in the presence of the longer spacer (that is 1,6-diaminoethane versus 1,2-diaminoethane) [27]. **VIII A** and **VIII B** showed LF values of 0.4 and 0.7, respectively, while for **VIII C**, the iodine LF was found to be 0.3 (Table 1, entries 3–5). The LF values found for **IV A**–**B** and **VIII A**–**C** were of the same order of magnitude, and higher than those previously reported for solid reagents based on the immobilization of IBX on both polymer resins and GO [14,20,25]. Moreover, the higher amount of Au with respect to iodine measured for **VIII A**–**C** proved that the initial linkage of mercapto containing spacers was not quantitative with respect to the Au binding sites available on the support (Table 2, entries 3–5).

### 3.4. Oxidation of Aromatic Alcohols with IV A–B and VIII A–C

**Scheme 4:**
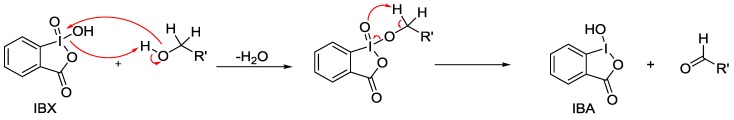
General mechanism of oxidation of primary alcohols with IBX.

**Scheme 5:**
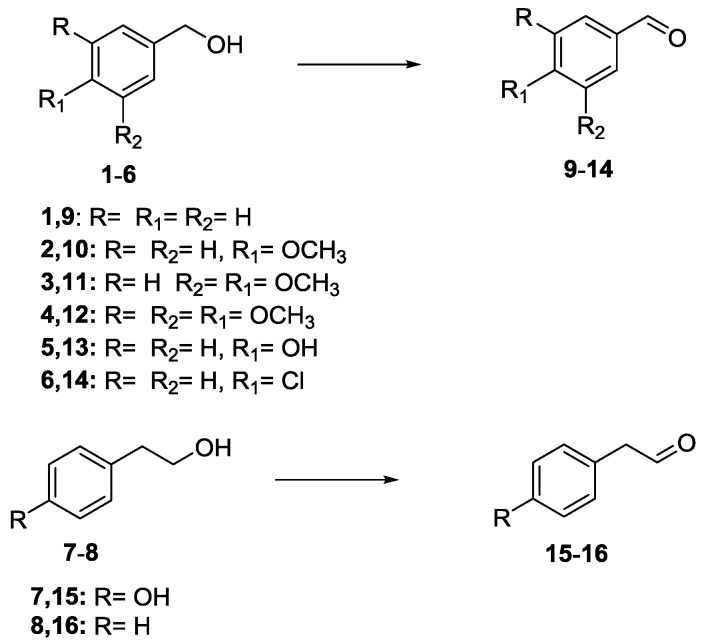
Oxidation of alcohols **1–8** with **IV A–B** and **VIII A–B**.

The mechanism of the oxidation of aromatic alcohols with IBX is reported in Scheme 4. The oxygen atom transfer from IBX to the substrate requires the initial addition of the alcohol on activated iodine followed by water elimination and disproportionation with the displacement of the aldehyde [14]. **IV A**–**B** and **VIII A**–**C** were applied for the oxidation of a large panel of aromatic alcohols, including benzyl alcohols **1**–**6** and phenethyl alcohols **7**,**8** (Scheme 5, Tables 4 and 5).

The reactions were performed treating the appropriate alcohol (1.0 mmol) with a slight excess of **IV A**–**B** and **VIII A**–**C** (1.2 IBX equivalent calculated on the basis of the specific LF value) in EtOAc (10 mL) at 80 °C for 24 h. Tentatively performing the oxidation in other reaction solvents usually applied for IBX transformations (e.g., Dimethyl Sulfoxide (DMSO) and water) were unsuccessful. 

Temperatures lower than c.a. 80 °C were not effective, while at temperatures higher than 80 °C, the reagents showed low stability affording only complex mixtures of reaction products. The reactions were analyzed by gas chromatography mass spectrometry (GC–MS) through a comparison with the original standards. Mass–to–charge ratio (m/z) values of aldehydes **9**–**16** are reported in Table 3 (the original MS fragmentation spectra are in Figure S1). Under optimal conditions, aromatic aldehydes **9**–**16** were detected as the only recovered products aside from unreacted substrates (Table 3 and Table 4). In the case of the oxidation of benzyl alcohol **1**, the reaction with commercially available IBX and with IBX supported on polystyrene (sIBX) were performed as references (Table 3, entries 1 and 2).

Homogeneous IBX showed a reactivity higher than the supported reagents in the oxidation of benzyl alcohol **1**, probably as a consequence of the diffusional barriers for the access of substrate to active iodine atom, with the only exception of **VIII-B**, which showed a comparable efficacy (Table 4, entry 1 versus entry 11). On one hand, **IV A**–**B** and **VIII A**–**B** oxidized benzyl alcohol **1** to aldehyde **9** in a higher yield with respect to sIBX, suggesting the beneficial role of MWCNTs as support with respect to the organic resin (Table 4 and Table 5). Irrespective of the experimental conditions, **VIII-C** was totally ineffective in the oxidation of **1**, and was not further investigated (Table 5, entry 19). Probably, the low reactivity of **VIII-C** was ascribable to the detrimental effect of the ester linkage with respect to the amide counterpart on the stability of the Iodine (V) active species [28]. As a general trend, benzyl alcohol derivatives **1**–**6** were more reactive than phenethyl alcohols **7**,**8**. Moreover, benzyl alcohol bearing electron donating substituents **2**–**5** were more reactive than **1** (Table 4 and Table 5), in accordance with previously reported data focusing on the role of the electron density on the benzylic position in the rate-determining step of IBX-mediated oxidations [14].

The dimension of the spacer also played a significant role, where **IV-B** and **VIII-B** bearing the longer spacer chains were the most reactive systems. The effect of the spacer on the reactivity of the supported reagents has been previously investigated, the increase of the length of the chains always being related to the increase of the low energy conformational changes attained by the reagent and to the reduction of the diffusional barrier for the substrates [29]. Finally, Au–MWCNTs based reagents **VIII A**–**B** were generally more reactive than the MWCNTs counterparts **IV A**–**B**, most likely due to the increased electron-transfer properties of the support as a consequence of the increased conductance of nanotubes in the Au carbon junctions [30]. The recyclability of supported IBX was evaluated for the more reactive **VIII B** reagent in the oxidation of benzylic alcohol **1**. After the first run, the reagent was recovered by filtration, washed with EtOAc, dried and restored in the active form by treatment with Oxone^®^ and methansulfonic acid. **VIII B** retained the same reactivity to afford aldehyde **9** in a quantitative yield for at least five successive runs. The absence of leaching of IBX from **VIII B** was confirmed by testing the oxidative capacity of the organic solution recovered after filtration of its EtOAc solution once maintained at reflux under the same experimental conditions applied for the oxidation. Any oxidation capacity was observed.

Compounds **IV B** and **VIII B** retained their morphological structural integrity after the oxidation of alcohol **1**, as highlighted by the TEM analysis of the recovered samples (Figure 1, panels B and D, respectively).

## 4. Conclusions

The preparation of a series of IBX based reagents supported on MWCNTs, as heterogeneous biomimetic systems for the selective oxidation of primary alcohols to the corresponding aldehydes under mild conditions, has been described. Two different types of carbon structures have been investigated, namely oxidized MWCNTs or an alternative, Au-decorated MWCNTs. The immobilization of the iodine active reagent was realized by exploiting the direct formation of an amide linkage with IBX, mediated by different spacer lengths, or through the high binding affinity of sulfur containing linkers in the case of the Au-decorated MWCNTs. In general, the benzyl alcohol derivatives were shown to be more active than the corresponding phenethyl alcohols, thus confirming the prominent role exerted by the electron density on the benzylic carbon in the rate-determining step of the IBX-mediated oxidative process [29]. In accordance with this hypothesis, benzyl alcohol bearing electron donating substituents showed the highest reactivity. The dimension of the spacer incorporated between the IBX fragment and the carbon nanotube surface also played a significant role. Indeed, the reagents bearing the longer spacer chains showed higher LF values and better oxidation performances. Regarding the LF values, the longer spacer may reduce steric hindrances for the IBA-functionalization of MWCNTs, increasing the number of groups involved in the multipoint covalent attachment [31]. Similarly, the better oxidation performance measured in the presence of the longer spacer was in accordance with previously reported data on the role that the spacer length can play for a certain mobility of the active species [32]. Interestingly, Au–MWCNTs based systems behaved as the more reactive reagents, thus justifying their increased electron-transfer properties ascribable to the presence of electroactive Au-carbon joints [33] in comparison with simple MWCNTs counterparts. The novel IBX supported reagents were easily recoverable from the reaction mixture, being successfully used for more runs after a simple reaction with the primary oxidant. These novel reagents can be applied in large scale processes, overcoming drawbacks associated with the use of oxidizing enzymes. Moreover, their metal-free structure, associated with the biocompatibility of MWCNTs, ensures novel reagents high eco-compatibility and low environmental impact. 

## Figures and Tables

**Table 1:** Electron binding energies (eV) of the showed element for the XPS analyzed **III B**, **IV B**, **VII A** and **VIII A** compounds.

Element	Reagent				Assignments
	III B	IV B	VII A	VIII A	
**N 1s**	400.5	400.5	400.5	400.5	N–H (amide)
**S 2p_3/2_**	-	-	164.4	-	C–S–C (sulfide)
			168.6	168.6	C–SO_x_–C
**Au 4f_7/2_**	-	-	84.0	84.0	Au-Au
**I 3d_5/2_**	619.2	-	619.2	-	I_2_
	621.5	621.5	621.5	621.5	I-O

**Table 2:** ICP–MS analyses of **IV A–B** and **VIII A–C** reagents.

Entry	Compound	ICP-MS^c^		LF ^a^
		Iodine (%)	Gold (%)	
1	**IV A**	0.03	-	0.3
2	**IV B**	0.21	-	2.1
3	**VIII A**	0.04	0.38	0.4
4	**VIII B**	0.07	4.7	0.7
5	**VIII C**	0.03	2.10	0.3

^a^ Loading Factor (LF) defined as mmol of iodine per gram of support.

**Table 3:** Mass–to–charge ratio (*m/z*) value and the abundance of mass spectra peaks of compounds **9–16**.

Products ^a^	*m/z* (%)
Benzaldehyde **(9)**	107 (10) [M+1], 106 (80) [M], 105 (72) (M-1)
4 Methoxy Benzaldehyde **(10)**	136 (20) [M], 135 (69) [M-1], 134 (100) [M-2], 133 (95) [M-3], 132 (77) [M-4], 131 (95) [M-5]
3-4 Dimethoxy Benzaldehyde **(11)**	167 (10) [M+1], 166 (52) [M], 165 (80) [M-1], 164 (92) [M-2], 163 (99) [M-3], 162 (87) [M-4], 161 (50) [M-5], 160 (17) [M-6]
3-4-5 Trimethoxy Benzaldehyde **(12)**	197 (2) [M+1], 196 (85)
4 HydroxyBenzaldehyde **(13)**	124 (2) [M+2], 123 (1) [M+1], 122 (100) [M], 121 (63) [M-1], 120 (10) [M-2]
4 Chlorobenzaldehyde **(14)**	142 (10) [M+2], 141 (12) [M+1], 140 (123) [M], 139 (45) [M-1], 138 (90) [M-2], 137 (50) [M-3], 136 (100) [M-4], 135 (1) [M-5]
4-Hydroxyphenylacetaldehyde **(15)**	136 (100)
Phenylacetaldehyde **(16)**	121 (2) [M+1], 120 (35) [M],

^a^ Mass spectroscopy was performed by using a GC–MS. The peak abundances reported in parentheses.

**Table 4:** Oxidation of compounds **1–8** with **IV A–B** ^a^.

Entry	Substrate	Oxidant	Product	Yield (%) ^b^
1	Benzyl alcohol **(1)**	**IBX**	**9**	95
2	Benzyl alcohol **(1)**	**sIBX ^c^**	**9**	25
3	Benzyl alcohol **(1)**	**IV A**	**9**	29
4	4-Methoxy benzyl alcohol **(2)**	**IV A**	**10**	35
5	3,4-Dimethoxy benzyl alcohol **(3)**	**IV A**	**11**	39
6	3,4,5-Trimethoxy benzy alcohol **(4)**	**IV A**	**12**	41
7	4-Hydroxy benzyl alcohol **(5)**	**IV A**	**13**	50
8	4-Chloro benzyl alcohol **(6)**	**IV A**	**14**	18
9	Tyrosol **(7)**	**IV A**	**15**	5
10	Phenethyl alcohol **(8)**	**IV A**	**16**	7
11	Benzyl alcohol **(1)**	**IV B**	**9**	52
12	4-Methoxy benzyl alcohol **(2)**	**IV B**	**10**	60
13	3,4-Dimethoxy benzyl alcohol **(3)**	**IV B**	**11**	62
14	3,4,5-Trimethoxy benzyl alcohol **(4)**	**IV B**	**12**	63
15	4-Hydroxy benzyl alcohol **(5)**	**IV B**	**13**	80
16	4-Chloro benzyl alcohol **(6)**	**IV B**	**14**	45
17	Tyrosol **(7)**	**IV B**	**15**	10
18	Phenethyl alcohol **(8)**	**IV B**	**16**	15

^a^ The reactions were performed treating the appropriate alcohol (0.1 mmol) with a slight excess of IV A–B (1.2 IBX equivalent calculated on the basis of the specific L.F. value) in EtOAc (1.0 mL) at reflux for 24 h. ^b^ The substrate was selectively converted only to the corresponding aldehyde. The yield was evaluated using n-dodecane as the internal standard. The conversion of substrate corresponds to the yield of detected products. ^c^ IBX supported on polystyrene.

**Table 5:** Oxidation of compounds **1**–**8** with **VIII A–C** ^a^.

Entry	Substrate	Oxidant	Product	Yield (%) ^b^
1	Benzyl alcohol **(1)**	**IBX**	**9**	95
2	Benzyl alcohol **(1)**	**sIBX ^c^**	**9**	25
3	Benzyl alcohol **(1)**	**VIII A**	**9**	38
4	4-Methoxy benzyl alcohol **(2)**	**VIII A**	**10**	44
5	3,4-Dimethoxy benzyl alcohol **(3)**	**VIII A**	**11**	48
6	3,4,5-Trimethoxy benzy alcohol **(4)**	**VIII A**	**12**	50
7	4-Hydroxy benzyl alcohol **(5)**	**VIII A**	**13**	68
8	4-Chloro benzyl alcohol **(6)**	**VIII A**	**14**	27
9	Tyrosol **(7)**	**VIII A**	**15**	9
10	Phenethyl alcohol **(8)**	**VIII A**	**16**	10
11	Benzyl alcohol **(1)**	**VIII B**	**9**	98
12	4-Methoxy benzyl alcohol **(2)**	**VIII B**	**10**	>99
13	3,4-Dimethoxy benzyl alcohol **(3)**	**VIII B**	**11**	>99
14	3,4,5-Trimethoxy benzyl alcohol **(4)**	**VIII B**	**12**	>99
15	4-Hydroxy benzyl alcohol **(5)**	**VIII B**	**13**	>99
16	4-Chloro benzyl alcohol **(6)**	**VIII B**	**14**	95
17	Tyrosol **(7)**	**VIII B**	**15**	20
18	Phenethyl alcohol **(8)**	**VIII B**	**16**	24
19	Benzyl alcohol **(1)**	**VIII C**	**9**	<3
20	Benzyl alcohol **(1)**	**VIII B**	**9**	96 ^d^

^a^ The reactions were performed treating the appropriate alcohol (0.1 mmol) with a slight excess of **IV A–B** (1.2 IBX equivalent calculated on the basis of the specific L.F. value) in EtOAc (1.0 mL) at reflux for 24 h. ^b^ The substrate was selectively converted only to the corresponding aldehyde. The yield was evaluated using n-dodecane as the internal standard. The conversion of the substrate corresponds to the yield of the detected products. ^c^ IBX supported on polystyrene. ^d^ The recyclability of supported IBX was evaluated for the more reactive **VIII B** reagent in the oxidation of benzylic alcohol **1**, after filtration and treatment with Oxone^®^ and methansulfhonic acid. **VIII B** retained the same reactivity to afford aldehyde **9** in c.a. quantitative yield for at least five successive runs.

## Supplementary Materials

The following are available online at http://www.mdpi.com/2079-4991/8/7/516/s1, Figure S1: original mass spectra of compounds **9**–**16**; Figure S2: FTIR analysis of oxMWCNTs **I**; Figure S3: FTIR analysis of **II A**; Figure S4: FTIR analysis of **III A**; Figure S5: FTIR analysis of **IV A**; Figure S6: SEM Back Scattered Electrons (BSE) analysis of **VIII B**.
