# Supplementary material for: Iodoxybenzoic Acid Supported on Multi Walled Carbon Nanotubes as Biomimetic Environmental Friendly Oxidative Systems for the Oxidation of Alcohols to Aldehydes
Source: Nanomaterials (Basel). 2018 Jul 10;8(7):516. doi: 10.3390/nano8070516 (PMC6071043; doi:10.3390/nano8070516)
Supplement: Supplementary file 1 [file nanomaterials-08-00516-s001.pdf]

Supporting Information

Iodoxybenzoic Acid Supported on Multi Walled Carbon Nanotubes as Biomimetic Environmental Friendly Oxidative Systems for the Oxidation of Alcohols to Aldehydes

Bruno Mattia Bizzarri [1], Issam Abdalghani [2], Lorenzo Botta [1], Anna Rita Taddei [3], Stefano Nisi [4], Marco Ferrante [4,5], Maurizio Passacantando [2], Marcello Crucianelli [2,*] and Raffaele Saladino [1,*]

Benzaldehyde **(9)**

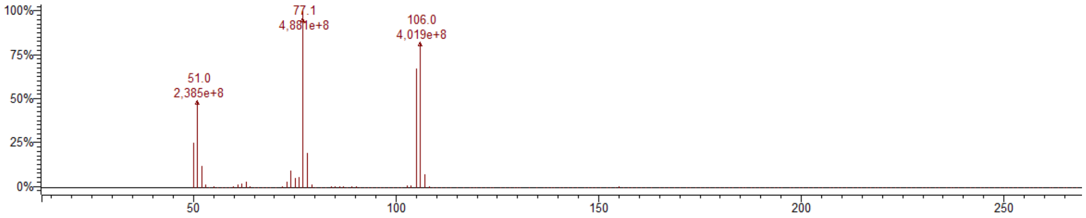

4 Methoxy Benzaldehyde **(10)**

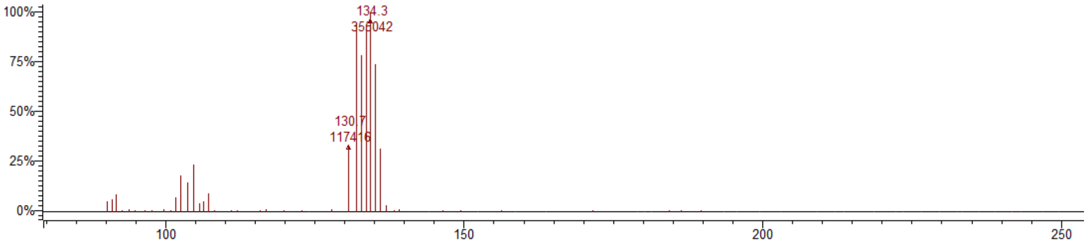

3-4 Dimethoxy Benzaldehyde **(11)**

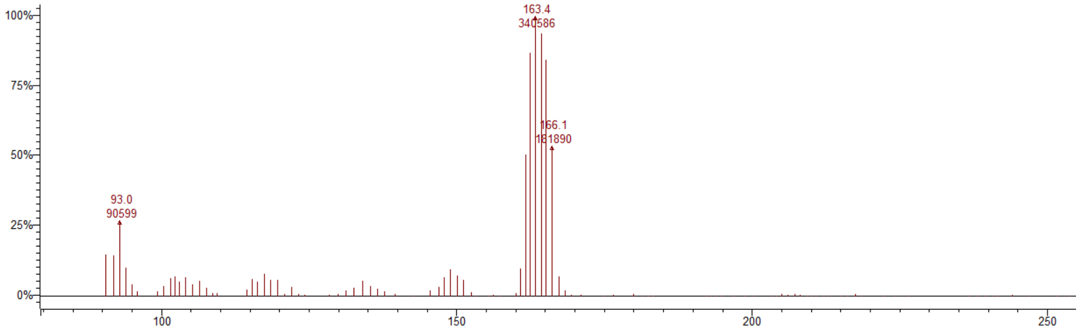

3-4-5 Trimethoxy Benzaldehyde **(12)**

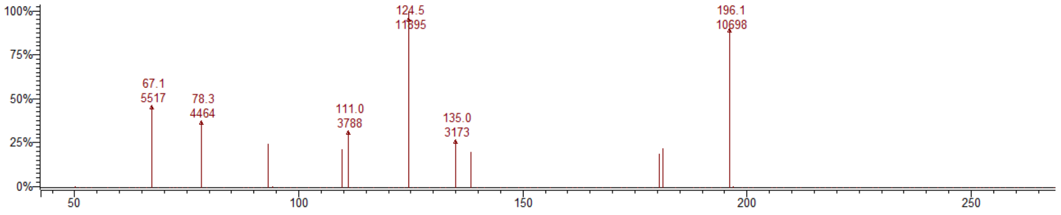

4 HydroxyBenzaldehyde **(13)**

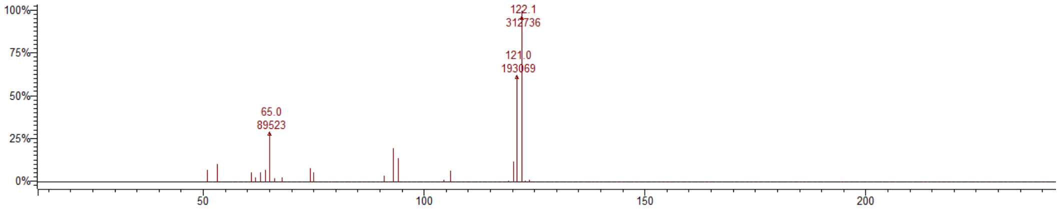

4 Chlorobenzaldehyde **(14)**

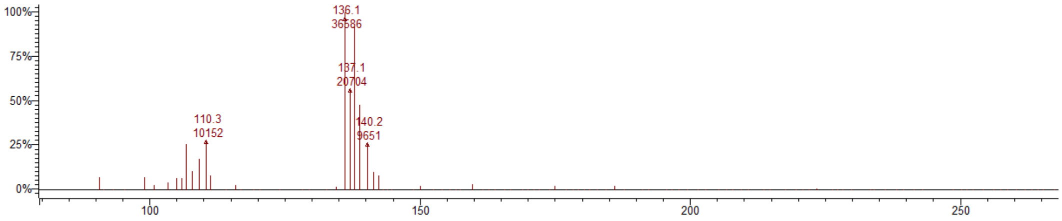

4-Hydroxyphenylacetaldehyde **(15)**

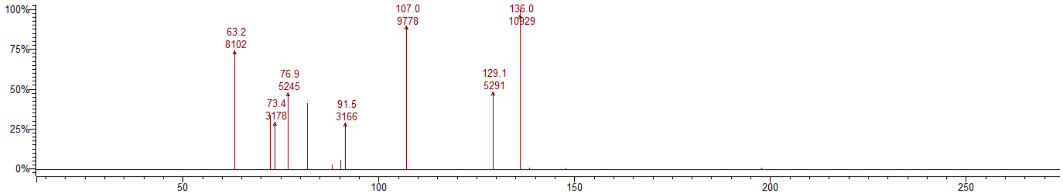

Phenylacetaldehyde **(16)**

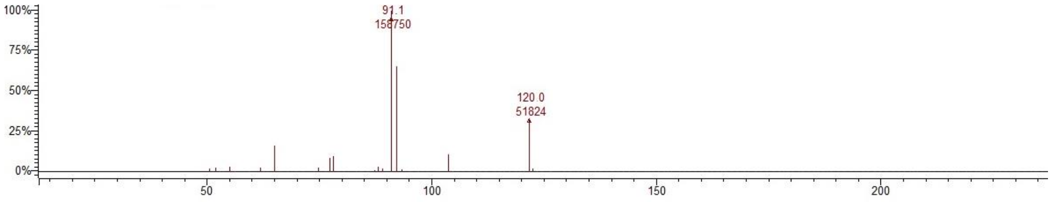

Figure S1. Mass fragmentation peaks of aldehydes (**9–16**).

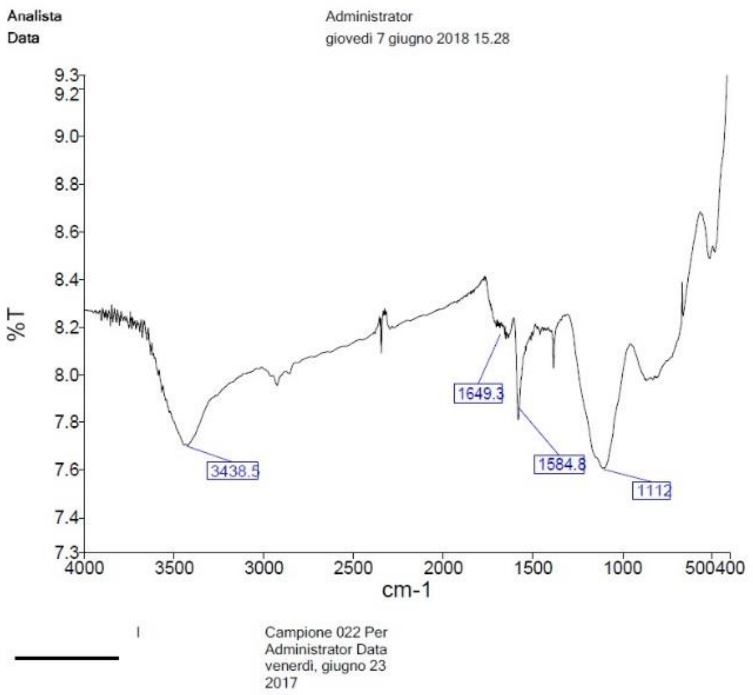

Figure S2. FT-IR analysis of oxMWCNTs **I**.

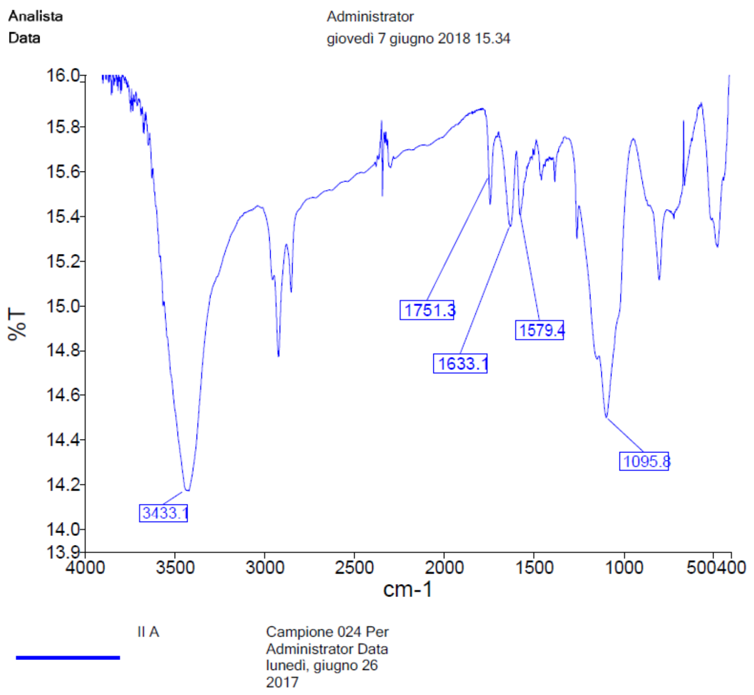

Figure S3. FT-IR analysis of **II-A.**

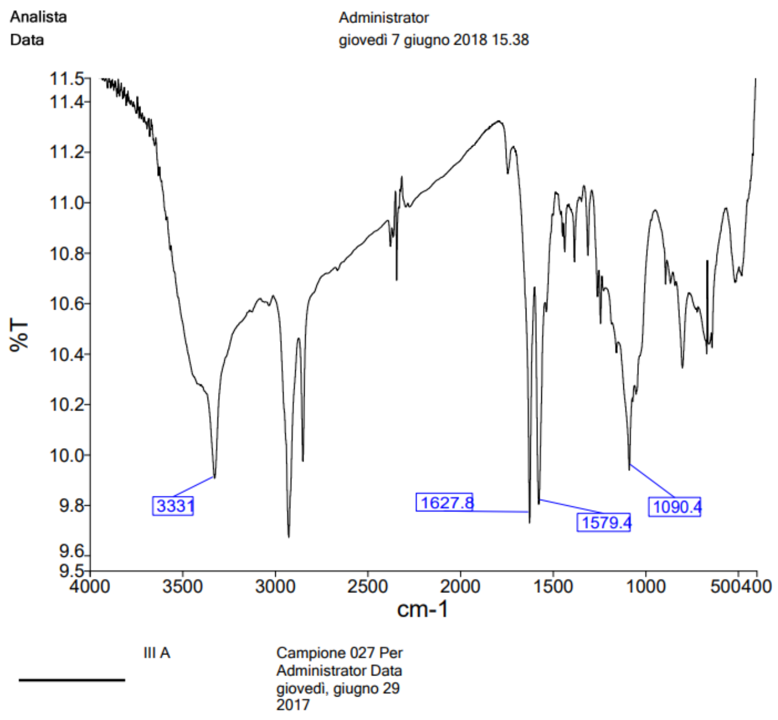

Figure S4. FT-IR analysis of **III-A**.

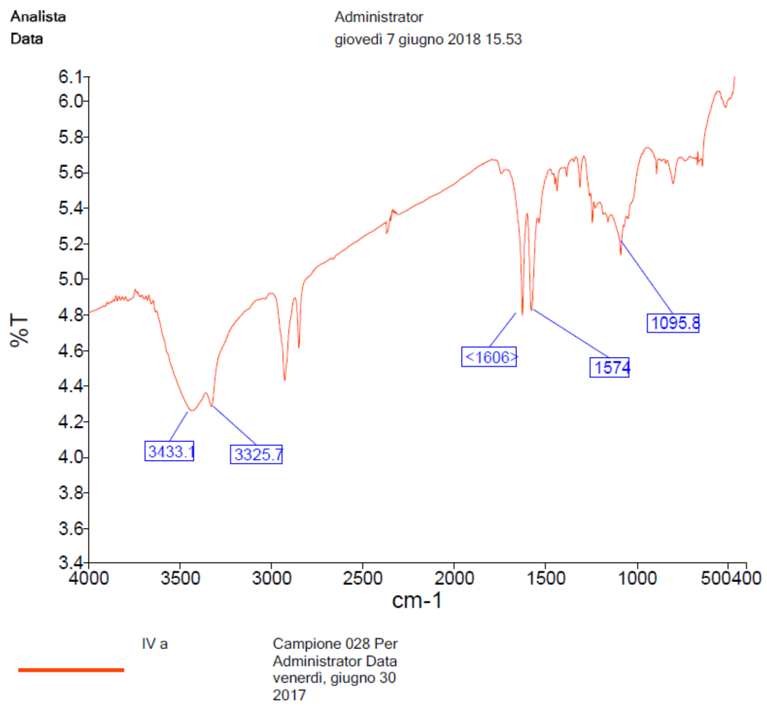

Figure S5. FT-IR analysis of **IV-A.**

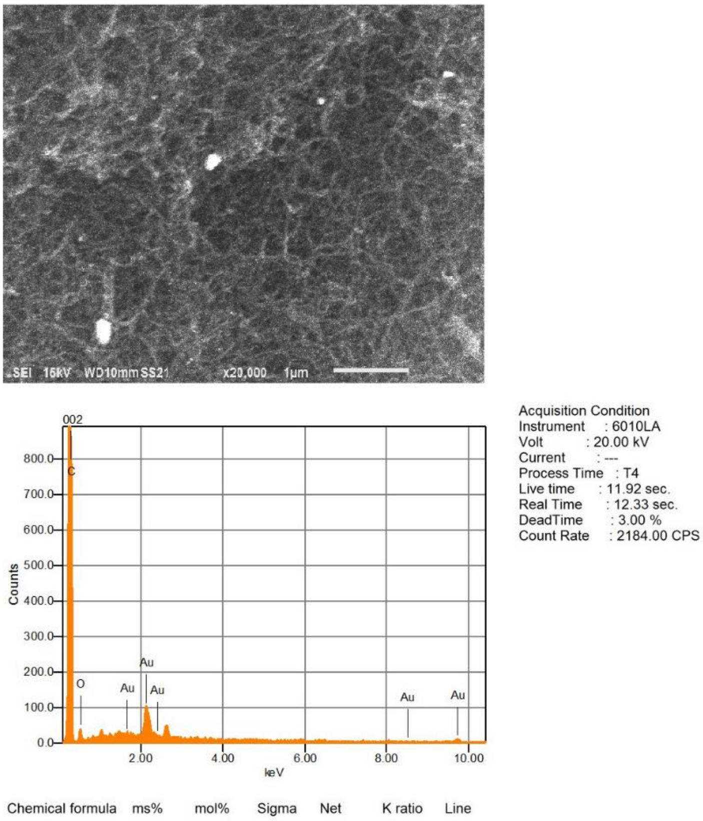

Figure S6. SEM Back Scattered Electrons (BSE) analysis of **VIII B.**
